# New York Academy of Medicine

**Published:** 1857-01

**Authors:** 


*New York Academy of Medicine. Special Meeting. The Opinions of Prof. E. H. Barton on Yellow Fever.*—Although it is hardly probable that we shall ever be called upon to meet yellow fever in our high northern latitude, yet it is daily becoming more evident that a single set of proximate causes govern most zymotic epidemics, and that the study of any one of them must throw light upon the others. The opinions of Dr. Barton, on yellow fever, are entitled to the highest consideration. We give the report of his remarks from the New York Daily Times:

"A special meeting of the New York Academy of Medicine was held at the University, on Wednesday evening last, Dr. Willard Parker, President, in the chair, who stated that the academy had been called together for the purpose of availing themselves of the opportunity to hear the views of Dr. Barton, of New Orleans, upon the subject of yellow fever. Dr. B. was well known to many as formerly connected with the school attached to the University, and as having, in New Orleans, in the Mexican war with Gen. Scott, had a large field for the observation and study of yellow fever, upon which he had read and written much. He was happy to introduce him to the academy.

"Dr. Barton briefly returned his thanks to the academy for the honor of appearing before them, and stated that a few years since he was appointed by the city of New Orleans, the chairman of a medical commission to examine and report upon the subject of yellow fever, in its relations to the city of New Orleans. That report was elaborate and voluminous, but to it he need not refer, for it had been published, and probably was in the hands of most of those present, and on the present occasion he should limit himself to the cause and prevention of this disease, without touching at all upon the treatment and many other points. How it originates, and how to prevent its occurrence, would be the subject of his evening's paper.

